# Description of movement sensor dataset for dog behavior classification

**DOI:** 10.1016/j.dib.2022.107822

**Published:** 2022-01-11

**Authors:** Antti Vehkaoja, Sanni Somppi, Heini Törnqvist, Anna Valldeoriola Cardó, Pekka Kumpulainen, Heli Väätäjä, Päivi Majaranta, Veikko Surakka, Miiamaaria V. Kujala, Outi Vainio

**Affiliations:** a Faculty of Medicine and Health Technology, Tampere University, P.O. Box 692, Tampere FI-33101, Finland; b Department of Equine and Small Animal Medicine, University of Helsinki, P.O. Box 57, Helsinki FI-00014, Finland; c Research Group for Emotions, Sociality, and Computing, Faculty of Information Technology and Communication Sciences, Tampere University, P.O. Box 100, Tampere FI-33014, Finland; d Master School, Lapland University of Applied Sciences, Jokiväylä 11 B, Rovaniemi 96300, Finland; e Department of Psychology, Faculty of Education and Psychology, University of Jyväskylä, P.O. Box 35, Jyväskylä FI-40014, Finland

**Keywords:** Dog activity classification, Behavior classification, Movement sensor, Accelerometer, Gyroscope, Machine learning

## Abstract

Movement sensor data from seven static and dynamic dog behaviors (sitting, standing, lying down, trotting, walking, playing, and (treat) searching i.e. sniffing) was collected from 45 middle to large sized dogs with six degree-of-freedom movement sensors attached to the collar and the harness. With 17 dogs the collection procedure was repeated. The duration of each of the seven behaviors was approximately three minutes. The order of the tasks was varied between the dogs and the two repetitions (for the 17 dogs). The behaviors were annotated post-hoc based on the video recordings made with two camcorders during the tests with one second resolution. The annotations were accurately synchronized with the raw movement sensors data.

The annotated data was originally used for training behavior classification machine learning algorithms for classifying the seven behaviors. The developed signal processing and classification algorithms are provided together with the raw measurement data and reference annotations. The description and results of the original investigation that the dataset relates to are found in: P. Kumpulainen, A. Valldeoriola Cardó, S. Somppi, H. Törnqvist, H. Väätäjä, P. Majaranta, Y. Gizatdinova, C. Hoog Antink, V. Surakka, M. V. Kujala, O. Vainio, A. Vehkaoja, Dog behavior classification with movement sensors placed on the harness and the collar, Applied Animal behavior Science, 241 (2021), 105,393.

## Specifications Table

**Table:** 

Subject	Applied Machine Learning
Specific subject area	Animal behavior classification based on movement sensor data
Type of data	Time series sensor data Table Signal processing code
How data were acquired	Movement sensors and visual reference video annotations Instruments: ActiGraph GT9X Link 6 Degree-of-Freedom movement sensors Panasonic HDC-SD600 and Sony HDR-CX450 video cameras Observer XT 10.5 video annotation software (Noldus, The Netherlands)
Data format	Raw movement sensor data Analyzed data (reference labeling by visual annotation of video recordings) Movement data analysis scripts
Parameters for data collection	6 degree-of-freedom (3D accelerometer and 3D gyroscope) movement sensors (ActiGraph GT9X Link) attached to the collar and the harness of the dog. Sampling rate was 100 Hz per channel. Also, true labels for the activities are provided based on post-hoc video annotations as well as the breed, gender, and age of the participant dogs.
Description of data collection	45 participating dogs were performing three static and four dynamic tasks, each task lasting for three minutes. Static tasks were: sitting, standing, lying down. Dynamic tasks were: trotting, walking, playing, and treat-searching (sniffing). The dogs were guided by their owner during the tasks. The order of the tasks was varied, and static and dynamic tasks were alternated. For 17 dogs also the data from the repetition of the procedure is available. The data collection procedures were video recorded and annotated post hoc.
Data source location	Institution: University of Helsinki City/Town/Region: Helsinki Country: Finland
Data accessibility	Repository name: Mendeley Data Data identification number: 10.17632/vxhx934tbn.2 Direct URL to data: http://dx.doi.org/10.17632/vxhx934tbn.2
Related research article	P. Kumpulainen, A. Valldeoriola Cardó, S. Somppi, H. Törnqvist, H. Väätäjä, P. Majaranta, Y. Gizatdinova, C. Hoog Antink, V. Surakka, M. V. Kujala, O. Vainio, A. Vehkaoja, Dog behavior classification with movement sensors placed on the harness and the collar, Applied Animal behavior Science, 241 (2021), 105,393. 10.1016/j.applanim.2021.105393

## Value of the Data

• The data is important because it enables researchers to develop methods for classifying the activities and behaviors of dogs. The developed methods can also be used to study the behavior of similar wild animals.
• The data set can benefit animal researchers for development of more automatic classification and detection of certain types of behavior and/or movement of animals.
• The data set provides a relatively large sample size of this type, with test-retest data available from part of the sample.
• The accelerometer data is measured simultaneously from two separate locations, neck collar and back of the harness, which enables comparison of the locations and velocity profiles manifested in each of these.
• The accelerometer data measured from two locations (neck and back) can be used for gaining accurate information from the dog's wellbeing and health, for example developing more efficient methods to detect stress or pain in dogs.
• The data includes both stationary position shifts and different locomotion examples, as well as the sniffing behavior of dogs.
• The data can benefit computer scientist who develop new methods for data analysis as well as animal researchers who can utilize the developed methods in their work.
• The data and the published data analysis algorithms can be directly used for further development of the classification algorithms. The data and classification algorithms published along with the data can be used for developing solutions for studying dog behavior as well as behavior of similar free-ranging wild animals.

## 1 Data Description

The dataset consists of following documents and files:

1. Data description.txt: Text document describing the contents of the other files in the repository.
2. DogInfo.csv: Comma separated values document containing following information about the dogs participating in the study: breed, weight, age, gender, and neutering status.
3. AnalysisCode.zip: Signal processing algorithm package for Matlab containing the codes needed to re-produce the analysis and the instructions for running the code and for making the required modifications to some of the functions in Statistic and Machine Learning Toolbox of Matlab.
4. DogMoveData.mat: Matlab data file containing one table-type variable that has following columns:
  ○ DogID (Number of the dog),
  ○ TestNum (Number of the test {1, 2}),
  ○ sensorData: Cell array of tables. Each table contains the measurement data from one recorded test at 100 Hz sampling rate. The descriptions of the columns of sensorData are provided in Table 1.

**Table 1:** Descriptions of the columns of sensorData cell array.

Column	Data type	Description
ABack	double n-by-3 matrix	Accelerator measurement from the sensor in the back. A matrix with three columns for x, y and z directions.
ANeck	double n-by-3 matrix	Accelerator measurement from the sensor in the neck. A matrix with three columns for x, y and z directions.
GBack	double n-by-3 matrix	Gyroscope measurement from the sensor in the back. A matrix with three columns for x, y and z directions.
GNeck	double n-by-3 matrix	Gyroscope measurement from the sensor in the neck. A matrix with three columns for x, y and z directions.
task	categorical n-by-1 array	the task given at the time, <undefined> when no task is being performed.
behavior	categorical n-by-3 array	three column array of the annotated behavior, maximum of three simultaneous annotations at the same time
PointEvent	categorical n-by-1 array	Short events annotated separately, Bark for example.
5. DogMoveData_csv_format.zip: Compressed package containing csv data file with the same content than in the .mat-datafile but arranged as 10,611,068 rows and 20 columns. The descriptions of the columns of DogMoveData.csv file are provided in Table 2.

**Table 2:** Descriptions of the columns of DogMoveData.csv file.

Column	Description
DogID	Number ID of the dog
TestNum	Number of the test {1, 2}
t_sec	Time from the start of the test in seconds
ABack_x	Accelerometer measurement from the sensor in the back, x-axis
ABack_y	Accelerometer measurement from the sensor in the back, y-axis
ABack_z	Accelerometer measurement from the sensor in the back, z-axis
ANeck_x	Accelerometer measurement from the sensor in the neck, x-axis
ANeck_y	Accelerometer measurement from the sensor in the neck, y-axis
ANeck_z	Accelerometer measurement from the sensor in the neck, z-axis
GBack_x	Gyroscope measurement from the sensor in the back, x-axis
GBack_y	Gyroscope measurement from the sensor in the back, y-axis
GBack_z	Gyroscope measurement from the sensor in the back, z-axis
GNeck_x	Gyroscope measurement from the sensor in the neck, x-axis
GNeck_y	Gyroscope measurement from the sensor in the neck, y-axis
GNeck_z	Gyroscope measurement from the sensor in the neck, z-axis
task	the task given at the time, <undefined> when no task is being performed
behavior_1	annotated behavior 1, maximum of three simultaneous annotations at the same time
behavior_2	annotated behavior 2, maximum of three simultaneous annotations at the same time
behavior_3	annotated behavior 3, maximum of three simultaneous annotations at the same time
PointEvent	Short events annotated separately, Bark for example

## 2 Experimental Design, Materials and Methods

### 2.1 Participants

In total 45 middle or large -sized dogs participated to the study. The dogs represented 27 different breeds. All the dogs were pet dogs and without major health problems that could have affected their physical performance. Table 3 presents the distribution of breed, weight and height information of the participating dogs. The average age was 4.9 years and the average weight 24.5 kg.

**Table 3:** Characteristics of the participant dogs [1].

Breed	Number	Weight (kg)	Age (years)
Australian Kelpie	1	18	3
Beauce Shepherd	3	30.33 (28–35)	3 (3–3)
Belgian Shepherd	1	29	6
Belgian Shepherd Groenendael	1	20	5
Belgian Shepherd Malinois	1	25	3
Border Collie	4	16.5 (15–20)	3.75 (3–5)
Bouvier des Flandres	1	30	7
Bouvier des Ardennes	2	22.5 (22–23)	4.5 (4–5)
Bull Terrier (Miniature)	1	17	2
Crossbreed	4	16.25 (13–20)	4.5 (3–7)
Dutch Shepherd	2	24 (23–25)	3 (3–3)
English Springer Spaniel	1	25	4
Finnish Lapphund	1	26	5
Flat-Coated Retriever	1	28	4
German Shepherd	3	32.33 (30–35)	3 (3–3)
Golden Retriever	3	30 (23–39)	4.67 (4–5)
Hovawart	2	34.5 (28–41)	5 (5–5)
Labrador Retriever	3	30 (23–37)	3 (3–3)
Lagotto Romagnolo	1	14	7
Lapponian Herder	2	21 (20–22)	3 (3–3)
Mudi	1	16	7
Nova Scotia Duck Tolling Retriever	1	20	5
Smooth Collie	1	18	3
Spanish Water Dog	2	22.5 (20–25)	7 (7–7)
Standard Poodle	1	31	7
Hungarian Vizsla	1	25	2

Middle to large -sized dogs were selected for the study sample, since extreme variations in dog size may affect the velocity profiles present in the data, especially in less controlled locomotion [4]. The dog breeds represented in the sample were found to provide an adequate sample for the application of this kind of analysis methods. It should be noted that the analysis algorithms are fitted for middle to large –sized dogs, which should be taken into account in the further application of the analysis algorithms, especially utilization for extremely small or extremely large dogs in free behavior -conditions.

### 2.2 Raw data collection

The experiments were conducted at the University of Helsinki, Faculty of Veterinary Medicine. The measurements were performed in a dog sporting hall in a testing area sized 10 m by 18 m covered with artificial turf. The test contained of seven tasks and dog owner was instructed to guide the dog through the tasks. The tasks were: sitting, standing, lying down, trotting, walking, playing, and treat-searching (sniffing), the three first ones being static and the four latter one dynamic tasks. Each task lasted for three minutes.

Dogs performed tasks sequentially and the order of the static and dynamic tasks was alternating. Treat search was always performed as the final task. It consisted of searching small pieces of dry dog food spread on the ground (area of 4 × 4 m) by sniffing. The whole procedure was performed two times and the order of the tasks was changed between the repeats.

Dogs were wearing two ActiGraph GT9X Link (ActiGraph LLC, Florida, USA) movement sensors including 3-axis accelerometer and 3-axis gyroscope sensors. Sampling rate was set at 100 Hz per sensor channel. One sensor was placed on the back belt of the dog's harness and the other sensor firmly on the ventral side of the neck collar. The sensors were attached so that the orientation of the sensors with respect to the dog was maintained throughout the test procedure. Dogs were on a 1.5 m leash and were led by their owners or the experimenter. The leash was connected to a separate collar than the movement sensor. The leash collar was placed closer to the dogs’ body than the movement sensor collar to minimize the former one from interfering the measurement signals. The dogs were given food rewards during the test to keep them focused and collaborative.

### 2.3 Annotation

The correct behavior of the dogs during the assigned tasks were annotated post-hoc based on video recordings made during the tests. The test procedure was recorded with two video cameras, Panasonic HDC-SD600 and Sony HDR-CX450. The cameras were positioned on the opposite walls of the test area. Observer XT 10.5 software by Noldus (The Netherlands), was used in the post-hoc annotation of the video recordings.

Minimum length of any behavior to be annotated was one second. Dynamic behaviors (Walking, Trotting, Galloping, Sniffing) were annotated only if being unambiguous. The criteria for annotation was that there was only one obvious and continuous dynamic behavior during which the dog was not leaning towards the handler or pulling the leash. These criteria were made to avoid the leaning or puling from affecting the gait pattern or the body orientation of the dog and such data from being included into the dataset. Galloping was annotated only during the playing task and sniffing only during the treat search task. Static behaviors or postures, i.e. Lying on chest, Sitting, and Standing were annotated when limbs did not move and there was no physical contact between the handler and the dog. Giving a treat was allowed during the static tasks and did not affect the annotations. Also other behaviors, such as drinking and shaking were annotated and are available in the data set but those have not been used in the studies reported in [1] or [3]. The total duration of the data is 106,110 s, i.e. 29.48 h. Situations where two or three behaviors are occurring simultaneously have been annotated accordingly but those have not been used in the analysis. The amount of data with two and three simultaneous annotation is 24,864 and 15,214 s, respectively. The dataset also includes significant amount, in total 34.4% or 36,494 s of data that has no annotation and is marked as undefined. These sections should not be used in developing supervised classification algorithms because they may also include targeted behaviors but are without labeling. behaviors Table 4 presents the descriptions of the behaviors considered in [1,3] and the amount of data of each behavior with no overlapping other annotated behavior. These descriptions were also the rules used in the annotations.

**Table 4:** Ethogram of the behaviors included in the statistical analyses [1].

Behavior	Description
Galloping	3- or 4-beat gait where the dog lifts and puts down both front and rear extremities in a coordinated manner, in 1–2–3-beat gait (canter) or in 1–2–3–4 beat gait (gallop). All four extremities are simultaneously in the air at some point in every stride. Galloping occurred only during Playing task. The total length of galloping as the only annotated behavior is 37 s.
Lying on chest	The dog's torso is touching the ground and hips are in the same level as shoulders. The dog can change balance point without using limbs. Total length: 4633 s.
Sitting	The dog has four extremities and rump on the ground. The dog can change balance point from central to hip or vice versa. Total length: 3895 s.
Sniffing	The dog has its head below its back line and moves its muzzle close to the ground. The dog walks, stands or performs another slow movement, but its chest and bottom do not touch the ground. Taking food from the ground and eating it can be included (eating was not coded separately). Total length: 10,262 s.
Standing	The dog has the four extremities on the ground, without the dog's torso touching the ground. Total length: 3709 s.
Trotting	2-beat gait where the dog lifts and puts down extremities in diagonal pairs at a speed faster than walking. Total length: 7174 s.
Walking	4-beat gait where the dog moves extremities at slow speed, legs are moved one by one in the order: left hind leg, left front leg, right hind leg, and right front leg. The dog moves straight forward or at maximum in 45° angle. Total length: 7503 s.

### 2.4 Dataset

The dataset is stored in Mendeley Data with the name Movement Sensor Dataset for Dog behavior Classification [2]. Two research papers [1,3] have been published utilizing the dataset. The data analysis scripts used to produce the results presented in [1] is included into the dataset. The scripts include codes data segmentation, feature extraction, feature selection and classification with four basic classifiers including leave-one-dog-out cross validation. The included classifiers are: linear discriminant analysis, quadrature discriminant analysis, support vector machine, and decision tree. The data analysis has been made with Matlab R2021b and running the code requires, besides the basic Matlab, also Statistic and Machine Learning Toolbox. Few functions in the toolbox have also been modified to allow the used cross validation scheme. Instructions for the modifications are included into the dataset.

## Ethics Statement

The study protocol was reviewed and accepted by the Ethical Committee for the Use of Animals in Experiments at the University of Helsinki (minutes 5/2017). All dog owners signed an informed consent before participating in the study. The attendants were free to cancel their participation at any time without giving a reason. No laboratory animal tests were performed.

## CRediT authorship contribution statement

**Antti Vehkaoja:** Conceptualization, Methodology, Software, Formal analysis, Data curation, Writing – original draft, Funding acquisition. **Sanni Somppi:** Conceptualization, Methodology, Investigation, Writing – review & editing. **Heini Törnqvist:** Conceptualization, Methodology, Investigation, Writing – review & editing. **Anna Valldeoriola Cardó:** Methodology, Investigation, Writing – review & editing. **Pekka Kumpulainen:** Conceptualization, Methodology, Software, Formal analysis, Data curation, Writing – review & editing. **Heli Väätäjä:** Writing – review & editing. **Päivi Majaranta:** Conceptualization, Writing – review & editing, Funding acquisition. **Veikko Surakka:** Conceptualization, Writing – review & editing, Funding acquisition. **Miiamaaria V. Kujala:** Conceptualization, Methodology, Writing – review & editing. **Outi Vainio:** Conceptualization, Writing – review & editing, Funding acquisition.

## Declaration of Competing Interest

The authors declare that they have no known competing financial interests or personal relationships which have or could be perceived to have influenced the work reported in this article.
